# Socioeconomic differences among community-dwelling diabetic adults screened for diabetic retinopathy and nephropathy: The 2015 Korean Community Health Survey

**DOI:** 10.1371/journal.pone.0191496

**Published:** 2018-01-24

**Authors:** Young-Hoon Lee

**Affiliations:** 1 Department of Preventive Medicine & Institute of Wonkwang Medical Science, Wonkwang University School of Medicine, Iksan, Jeonbuk, Republic of Korea; 2 Regional Cardiocerebrovascular Center, Wonkwang University Hospital, Iksan, Jeonbuk, Republic of Korea; Universidad Miguel Hernandez de Elche, SPAIN

## Abstract

We investigated the association between socioeconomic status (SES) and screening for diabetic retinopathy (DR) and diabetic nephropathy (DN) in community-dwelling diabetics. We analyzed data from 22,134 people with diabetes aged ≥19 years at the time of the nationwide 2015 Korean Community Health Survey. Multiple logistic regression analysis was used to explore the relationship between SES and screening for DR and DN both before and after adjustment for health behaviors, comorbidities, and educational level. Of all diabetic subjects, 33.9% and 38.1% underwent DR and DN screening, respectively. In the fully adjusted model, the extent of the DR and DN screening trended significantly lower as the educational level fell. Monthly household income was positively associated with DR screening, but a lower odds ratio (OR) for DN screening was evident only when the lowest and highest income groups were compared. Compared with managers/professionals, agricultural/forestry/fishery workers (OR 0.81, 95% confidence interval [CI] 0.69–0.96) and mechanical/manual laborers (OR 0.83, 95% CI 0.71–0.97) had lower ORs for DN screening. Residents in rural (compared with urban) areas and widows/widowers (compared with members of couples) were significantly less likely to undergo screening for DR and DN. Similar findings were obtained when the analysis was limited to those who had been educated about diabetes. In conclusion, socioeconomic inequalities were evident in terms of screening for DR and DN in community-dwelling Korean diabetics, regardless of whether they had reported receiving diabetes education. Tailored public health policies (and societal attention) are required to aid the socioeconomically disadvantaged.

## Introduction

Diabetes mellitus, one of the leading causes of death worldwide, is rapidly increasing in prevalence. Prevention of diabetic complications is important because such complications increase medical costs, lower quality of life, increase mortality, and increase social burdens [1–3]. Diabetic retinopathy (DR), a principal complication of diabetes, is a leading cause of preventable blindness and visual impairment [4]. If DR is detected early, blindness can be prevented using laser photocoagulation therapy [5]. Diabetic nephropathy (DN), one of the most frequent complications of diabetes, is a major cause of end-stage renal disease [6]. As early detection and appropriate treatment of diabetic complications, including DR and DN, can improve the prognosis of diabetic patients [7,8], it is very important to manage diabetic complications in a timely manner; to this end, regular routine screening is essential. An annual dilated fundus examination by an ophthalmologist and a urinary albumin level test scheduled by a physician are recommended for all patients with type 2 diabetes [9,10].

Recent Korean studies using National Health and Nutrition Survey data found that the screening rate for DR was 36.3–37.6% and that for DN was 40.5–46.1% [11,12]. Also, National Health Insurance data reveal that only 30% of diabetic patients underwent regular annual screening for DR (dilated fundus examination); the prevalence of DR in Korea has increased steadily [13]. Therefore, it is important to identify the characteristics of high-risk groups that do not undergo screening; the screening rate for diabetic complications in Korea is lower than that of other developed countries [14–16]. Previous studies explored factors that reduced DR and DN screening [11,12], but few studies on large populations have comprehensively examined whether socioeconomic inequalities are in play in Korea.

Therefore, we explored the association between socioeconomic status (SES) and screening for DR and DN in a large population of community-dwelling diabetics to assist public health policymakers. We also explored whether socioeconomic inequalities in terms of DR and DN screening were still evident among subjects who had been educated on management of diabetic complications.

## Methods

### Study population

We analyzed data from the 2015 Korean Community Health Survey (KCHS) conducted by the Korea Centers for Disease Control and Prevention. The KCHS is a nationwide survey conducted by trained surveyors, who performed personal interviews; all data are electronically stored. A sample of 900 participants from 253 community health centers was selected to achieve a sampling error of ±3% with a 95% confidence level for each main health index in each community health center. The KCHS utilized a two-stage sampling design and registered population data from the Ministry of Public Administration and Security. In the first stage, the sampling area (Tong/Ban/Ri), which is a primary sampling unit, was determined based on the number of households in the smallest administrative units (Dong/Eup/Myeon) using a sampling method with probability proportionate to size. In the second stage, the sample households were extracted from the sampling area using a systematic sampling method. All members of households over the age of 19 were interviewed. Using a multistage stratified cluster sampling procedure, the survey included a total of 228,558 household residents at least 19 years of age. Of the total participants, 22,937 had been diagnosed with diabetes. After excluding 803 participants for whom SES and covariate data were missing, the final sample consisted of 22,134 people with diabetes (10,797 males and 11,337 females). This study was conducted in accordance with the Declaration of Helsinki guidelines. Written informed consent was obtained from all participants of the KCHS. The study protocol was approved by the institutional review board of the Wonkwang University Hospital (WKUH 2017-05-019).

### Data

SES, health behavior, and health status were investigated using a standardized questionnaire. A detailed description of the independent variables is provided in Table 1. At the start of the investigation, 1,000 thousand Korean Won (KRW) was equivalent to 845.7 USD. The National Basic Livelihood Security (NBLS) system provides social assistance to low-income households in the form of livelihood grants, housing subsidies, education subsidies, and medical aid. People who had never smoked or had smoked less than 100 cigarettes in their lifetime were considered non-smokers. Former smokers were defined as those who had smoked at least 100 cigarettes in their lifetime but were not smokers at the time of the survey. Current smokers were defined as those who had smoked at least 100 cigarettes in their lifetime and who currently smoked every day or on some days. Average daily alcohol consumption (number of drinks/day) was calculated according to the frequency of alcohol consumption during the previous year and the average number of standard drinks of each beverage type consumed on a single occasion. Alcohol consumption was classified as none, ≤1 drink/day, >1 to 2 drinks/day, >2 to 4 drinks/day, or >4 drinks/day.

**Table 1:** Summary of independent variables.

Variables	Question	Category
Gender	What is your gender?	Male or female
Age group	What is your age?	19–29, 30–39, 40–49, 50–59, 60–69, or ≥70 years
Educational level	Where did you go to school? Did you graduate from school?	No formal education, primary school, middle school, high school, or college or higher
Monthly household income	What was your average monthly household income in the past year, including wages, real estate income, pensions, interest, government subsidies, and allowances for relatives or children?	≤1,000, 1,000–1,999, 2,000–2,999, 3,000–3,999, 4,000–4,999, or ≥5,000 thousand KRW
Occupation	What occupation are you currently engaged in?	Manager or professional, clerk, service or sales worker, agricultural/forestry/fishery worker, mechanical or manual laborer, or housewife/student
National Basic Livelihood Security status	Does your household currently receive National Basic Livelihood Security?	Recipient or non-recipient
Residence type	Is your residence urban or rural?	Urban or rural
Marital status	Have you ever been married (including a de facto marriage)? What is your current marital status? (1) Married and living together (2) Married but not living together (3) Widowed (4) Divorced	Never married, married, divorced/separated, or widowed
Smoking status	Have you smoked more than 5 packs (100 cigarettes) during your life? Do you smoke now? (1) I smoke every day (2) I smoke sometimes (3) I smoked in the past, but I do not smoke now	Non-, former, or current smoker
Alcohol consumption	Have you been drinking for the last year? How often do you drink alcohol? How much do you drink on a single occasion?	None, ≤1 drink/day, >1 to 2 drinks/day, >2–4 drinks/day, or >4 drinks/day
Walking activity	How many days did you walk for at least 10 minutes at a time in the last week?	≤4 or ≥5 days per week
Typical perceived stress	How often do you feel stressed in your usual life? (1) Very often (2) Often (3) Rarely (4) Almost never	Feeling often (1+2) or rarely (3+4)
Diagnosis of hypertension	Have you been diagnosed with hypertension?	Never or ever
Diagnosis of dyslipidemia	Have you been diagnosed with dyslipidemia?	Never or ever
Diabetes education	Have you ever been trained in the management of diabetes at a medical clinic, oriental medical clinic, or public health center?	Never or ever

The outcome variables were screening for DR and DN, and they were explored using the following questions: “Have you ever had an eye examination (fundoscopy) to see if diabetic eye complications occurred during the past year?”, and “Have you ever had a precise urine test (microalbuminuria) (with the exception of the stick test) to see if diabetic complications in the kidneys developed during the past year?”

### Statistical analysis

The numbers and percentages of screenings for DR and DN were determined for each general characteristic (gender, age, educational level, monthly household income, occupation, NBLS status, residence type, marital status, alcohol consumption, walking activity, perceived stress, diagnosis of hypertension, diagnosis of dyslipidemia, and diabetes education). Differences in the general characteristics among groups were compared using the chi-square test (nominal independent variables) or the Jonckheere–Terpstra test (ordinal independent variables). Multiple logistic regression analysis was employed to explore the association between SES and screening for DR and DN; we constructed three sequential models. Model 1 was unadjusted; Model 2 was adjusted for gender, age, and other socioeconomic variables; and Model 3 was Model 2 with the addition of smoking status, alcohol consumption, walking activity, perceived stress, diagnosis of hypertension or dyslipidemia, and diabetes education. Finally, the analysis was repeated using only data on subjects who had been educated about diabetes. The odds ratios (ORs) with 95% confidence intervals (CIs) of the effects of socioeconomic variables on DR and DN screening were calculated. All statistical analyses were performed with the aid of SPSS software version 22.0 (IBM Co., Armonk, NY, USA). A *P*-value <0.05 was considered to reflect statistical significance.

## Results

### Characteristics of study population

Table 2 lists the general characteristics of all subjects by DR and DN screening status. Of all 22,134 diabetics, 33.9% (7,508 subjects) underwent DR screening and 38.1% (8,431 subjects) underwent DN screening. The proportion undergoing both DR and DN screening was 24.2% (5,361 subjects).

**Table 2:** General characteristics of the study population according to diabetic retinopathy and nephropathy screening status (n = 22,134).

	*N (%)*	Diabetic retinopathy screening		*P*-value*	Diabetic nephropathy screening		*P*-value*
		Test (-)	Test (+)		Test (-)	Test (+)	
Total	*22*,*134 (100*.*0)*	14,626 (66.1)	7,508 (33.9)		13,703 (61.9)	8,431 (38.1)	
Gender				0.852			<0.001
Male	*10*,*797 (48*.*8)*	7,128 (66.0)	3,669 (34.0)		6,517 (60.4)	4,280 (39.6)	
Female	*11*,*337 (51*.*2)*	7,498 (66.1)	3,839 (33.9)		7,186 (63.4)	4,151 (36.6)	
Age, years				<0.001			<0.001
19–29	*83 (0*.*4)*	56 (67.5)	27 (32.5)		48 (57.8)	35 (42.2)	
30–39	*414 (1*.*9)*	303 (73.2)	111 (26.8)		249 (60.1)	165 (39.9)	
40–49	*1*,*671 (7*.*5)*	1,139 (68.2)	532 (31.8)		998 (59.7)	673 (40.3)	
50–59	*4*,*420 (20*.*0)*	2,895 (65.5)	1,525 (34.5)		2,582 (58.4)	1,838 (41.6)	
60–69	*6*,*590 (29*.*8)*	4,109 (62.4)	2,481 (37.6)		3,953 (60.0)	2,637 (40.0)	
≥70	*8*,*956 (40*.*5)*	6,124 (68.4)	2,832 (31.6)		5,873 (65.6)	3,083 (34.4)	
Educational level				<0.001			<0.001
No formal education	*4*,*675 (21*.*1)*	3,429 (73.3)	1,246 (26.7)		3,290 (70.4)	1,385 (29.6)	
Primary school	*6*,*071 (27*.*4)*	4,033 (66.4)	2,038 (33.6)		3,849 (63.4)	2,222 (36.6)	
Middle school	*3*,*496 (15*.*8)*	2,280 (65.2)	1,216 (34.8)		2,153 (61.6)	1,343 (38.4)	
High school	*5*,*195 (23*.*5)*	3,245 (62.5)	1,950 (37.5)		2,970 (57.2)	2,225 (42.8)	
College or higher	*2*,*697 (12*.*2)*	1,639 (60.8)	1,058 (39.2)		1,441 (53.4)	1,256 (46.6)	
Monthly household income, thousand KRW				<0.001			<0.001
<1,000 (<846 USD)	*8*,*367 (37*.*8)*	5,752 (68.7)	2,615 (31.3)		5,574 (66.6)	2,793 (33.4)	
1,000–1,999 (846–1,690 USD)	*4*,*864 (22*.*0)*	3,199 (65.8)	1,665 (34.2)		2,997 (61.6)	1,867 (38.4)	
2,000–2,999 (1,691–2,536 USD)	*3*,*485 (15*.*7)*	2,280 (65.4)	1,205 (34.6)		2,048 (58.8)	1,437 (41.2)	
3,000–3,999 (2,537–3,382 USD)	*2*,*232 (10*.*1)*	1,450 (65.0)	782 (35.0)		1,297 (58.1)	935 (41.9)	
4,000–4,999 (3,383–4,227 USD)	*1*,*383 (6*.*2)*	860 (62.2)	523 (37.8)		785 (56.8)	598 (43.2)	
≥5,000 (≥4,228 USD)	*1*,*803 (8*.*1)*	1,085 (60.2)	718 (39.8)		1,002 (55.6)	801 (44.4)	
Occupation				<0.001			<0.001
Manager and professional	*1*,*007 (4*.*5)*	634 (63.0)	373 (37.0)		529 (52.5)	478 (47.5)	
Clerk	*629 (2*.*8)*	393 (62.5)	236 (37.5)		353 (56.1)	276 (43.9)	
Service or sales worker	*1*,*870 (8*.*4)*	1,226 (65.6)	644 (34.4)		1,101 (58.9)	769 (41.1)	
Agricultural, forestry, or fishery worker	*3*,*713 (16*.*8)*	2,659 (71.6)	1,054 (28.4)		2,532 (68.2)	1,181 (31.8)	
Mechanical or manual laborer	*3*,*581 (16*.*2)*	2,447 (68.3)	1,134 (31.7)		2,254 (62.9)	1,327 (37.1)	
Housewife or student	*11*,*334 (51*.*2)*	7,267 (64.1)	4,067 (35.9)		6,934 (61.2)	4,400 (38.8)	
National Basic Livelihood Security status				0.504			0.027
Recipient	*1*,*789 (8*.*1)*	1,195 (66.8)	594 (33.2)		1,151 (64.3)	638 (35.7)	
Non-recipient	*20*,*345 (91*.*9)*	13,431 (66.0)	6,914 (34.0)		12,552 (61.7)	7,793 (38.3)	
Residence type				<0.001			<0.001
Rural	*11*,*737 (53*.*0)*	8,158 (69.5)	3,579 (30.5)		7,811 (66.6)	3,926 (33.4)	
Urban	*10*,*397 (47*.*0)*	6,468 (62.2)	3,929 (37.8)		5,892 (56.7)	4,505 (43.3)	
Marital status				<0.001			<0.001
Married	*15*,*470 (69*.*9)*	10,025 (64.8)	5,445 (35.2)		9,313 (60.2)	6,157 (39.8)	
Divorced or separated	*1*,*213 (5*.*5)*	812 (66.9)	401 (33.1)		732 (60.3)	481 (39.7)	
Widowed	*4*,*898 (22*.*1)*	3,412 (69.7)	1,486 (30.3)		3,328 (67.9)	1,570 (32.1)	
Never married	*553 (2*.*5)*	377 (68.2)	176 (31.8)		330 (59.7)	223 (40.3)	
Smoking status				<0.001			0.002
Non-smoker	*12*,*613 (57*.*0)*	8,255 (65.4)	4,358 (34.6)		7,886 (62.5)	4,727 (37.5)	
Former smoker	*5*,*911 (26*.*7)*	3,833 (64.8)	2,078 (35.2)		3,540 (59.9)	2,371 (40.1)	
Current smoker	*3*,*610 (16*.*3)*	2,538 (70.3)	1,072 (29.7)		2,277 (63.1)	1,333 (36.9)	
Alcohol consumption				<0.001			<0.001
None	*10*,*602 (47*.*9)*	6,867 (64.8)	3,735 (35.2)		6,545 (61.7)	4,057 (38.3)	
≤1 drink/day	*4*,*423 (20*.*0)*	2,858 (64.6)	1,565 (35.4)		2,701 (61.1)	1,722 (38.9)	
>1 to 2 drinks/day	*2*,*793 (12*.*6)*	1,808 (64.7)	985 (35.3)		1,668 (59.7)	1,125 (40.3)	
>2 to 4 drinks/day	*2*,*276 (10*.*3)*	1,573 (69.1)	703 (30.9)		1,408 (61.9)	868 (38.1)	
>4 drinks/day	*2*,*040 (9*.*2)*	1,520 (74.5)	520 (25.5)		1,381 (67.7)	659 (32.3)	
Walking activity, days/week				0.001			<0.001
≤ 4	*11*,*487 (51*.*9)*	7,707 (67.1)	3,780 (32.9)		7,305 (63.6)	4,182 (36.4)	
≥ 5	*10*,*647 (48*.*1)*	6,919 (65.0)	3,728 (35.0)		6,398 (60.1)	4,249 (39.9)	
Perceived stress				0.045			0.002
Feeling often	*16*,*400 (74*.*1)*	10,899 (66.5)	5,501 (33.5)		10,252 (62.5)	6,148 (37.5)	
Feeling rarely	*5*,*734 (25*.*9)*	3,727 (65.0)	2,007 (35.0)		3,451 (60.2)	2,283 (39.8)	
Diagnosed with hypertension				0.060			0.857
Never	*8*,*639 (39*.*0)*	5,644 (65.3)	2,995 (34.7)		5,342 (61.8)	3,297 (38.2)	
Ever	*13*,*495 (61*.*0)*	8,982 (66.6)	4,513 (33.4)		8,361 (62.0)	5,134 (38.0)	
Diagnosed with dyslipidemia				<0.001			<0.001
Never	*14*,*420 (65*.*1)*	9,942 (68.9)	4,478 (31.1)		9,451 (65.5)	4,969 (34.5)	
Ever	*7*,*714 (34*.*9)*	4,684 (60.7)	3,030 (39.3)		4,252 (55.1)	3,462 (44.9)	
Diabetes education received				<0.001			<0.001
Never	*15*,*950 (72*.*1)*	11,290 (70.8)	4,660 (29.2)		10,624 (66.6)	5,326 (33.4)	
Ever	*6*,*184 (27*.*9)*	3,336 (53.9)	2,848 (46.1)		3,079 (49.8)	3,105 (50.2)	

*Differences among groups were compared using the chi-square test (nominal independent variables) or the Jonckheere–Terpstra test (ordinal independent variables)

Significantly fewer females than males underwent DN screening; however, there was no significant gender difference in the proportions who had undergone DR screening. Participants aged 60–69 years (37.6%) and 19–29 years (42.2%) had the highest screening rates for DR and DN, respectively. Participants aged 30–39 years (26.8%) and ≥70 years (34.4%) had the lowest screening rates for DR and DN, respectively. People with higher educational levels and monthly household incomes had higher screening rates for DR and DN (*P* <0.001). Regarding specific occupations, screening rates for DR and DN were highest for clerks and managers/professionals, respectively. NBLS recipients were screened for DN significantly less often than were non-recipients; however, DR screening rates did not differ between recipients and non-recipients. Those who lived in urban regions walked more, had higher stress levels, were more likely to be diagnosed with dyslipidemia, reported receiving diabetes education more frequently, and had higher rates of DR and DN screening compared with the control groups. Significant differences in DR and DN screening rates based on marital status were observed; the highest DR screening rates were observed in married participants, while the highest DR screening rates were observed in those who had never married. Significant differences in DR and DN screening rates were observed based on smoking status; non-smokers had the highest and current smokers the lowest DR and DN screening rates. Further, those with higher levels of alcohol consumption had higher screening rates for both DR and DN (Table 2).

### Association of SES with DR and DN screening

Table 3 shows the relationships between SES and screening for both DR and DN. In Models 1, 2 and 3, ORs for both DR and DN screening decreased as function of educational level. After full adjustment (Model 3), those who had no formal versus a college or higher education had an OR for DR screening of 0.60 (95% CI, 0.52–0.68) and a OR for DN screening of 0.68 (95% CI, 0.60–0.78). In Model 1, ORs for both DR and DN screening decreased as a function of household income. In Models 2 and 3, as monthly household income fell, significant decreases in the ORs for DR screening were evident. However, in Models 2 and 3, only those with the lowest household income (<1,000 thousand KRW) had a lower OR for DN screening compared to those with the highest household income. Although the ORs for DR screening of agricultural/forestry/fishery workers and mechanical/manual laborers were significantly lower in Model 1, no significant effect of occupation on DR or DN screening was apparent in Models 2 and 3. In contrast, the ORs for DN screening of agricultural/forestry/fishery workers and mechanical/manual laborers were significantly lower in Models 1, 2, and 3: 0.81 (95% CI, 0.69–0.96) for agricultural/forestry/fishery workers and 0.83 (95% CI, 0.71–0.97) for mechanical/manual laborers in Model 3. We found no significant differences in the ORs for DR or DN screening by NBLS status after adjusting the variables, although the OR for DN screening of NBLS recipients was significantly lower in Model 1. In all Models, subjects living in rural areas had significantly lower ORs for DR and DN screening than those living in urban areas. In all Models, widows/widowers had significantly lower ORs for DR and DN screening than those whose spouses were alive: 0.90 (95% CI, 0.83–0.98) for DR and 0.86 (95% CI, 0.79–0.93) for DN in Model 3. However, there were no significant differences in the ORs for DR or DN screening among those who were divorced, separated, never married, or married.

**Table 3:** Relationships between socioeconomic status indicators and screening for diabetic retinopathy and nephropathy (n = 22,134).

	Diabetic retinopathy screening			Diabetic nephropathy screening		
	Model 1	Model 2	Model 3	Model 1	Model 2	Model 3
Educational level						
College or higher	1.00	1.00	1.00	1.00	1.00	1.00
High school	0.93 (0.85–1.02)	0.93 (0.84–1.03)	0.97 (0.87–1.08)	0.86 (0.78–0.94)	0.92 (0.83–1.01)	0.95 (0.86–1.05)
Middle school	0.83 (0.75–0.92)	0.78 (0.69–0.87)	0.83 (0.73–0.93)	0.72 (0.65–0.79)	0.77 (0.69–0.87)	0.82 (0.73–0.92)
Primary school	0.78 (0.71–0.86)	0.72 (0.64–0.81)	0.78 (0.69–0.88)	0.66 (0.60–0.73)	0.76 (0.68–0.85)	0.82 (0.73–0.92)
No formal education	0.56 (0.51–0.62)	0.52 (0.46–0.59)	0.60 (0.52–0.69)	0.48 (0.44–0.53)	0.60 (0.52–0.68)	0.68 (0.60–0.78)
Monthly household income, thousand KRW						
≥5,000 (≥4,228 USD)	1.00	1.00	1.00	1.00	1.00	1.00
4,000–4,999 (3,383–4,227 USD)	0.92 (0.80–1.06)	0.96 (0.83–1.11)	0.97 (0.84–1.13)	0.95 (0.83–1.10)	1.00 (0.86–1.15)	1.01 (0.87–1.17)
3,000–3,999 (2,537–3,382 USD)	0.82 (0.72–0.93)	0.86 (0.75–0.98)	0.88 (0.77–0.99)	0.90 (0.80–1.02)	0.98 (0.86–1.11)	1.00 (0.88–1.14)
2,000–2,999 (1,691–2,536 USD)	0.80 (0.71–0.90)	0.84 (0.75–0.95)	0.87 (0.76–0.98)	0.88 (0.78–0.99)	0.99 (0.88–1.11)	1.01 (0.90–1.14)
1,000–1,999 (846–1,690 USD)	0.79 (0.70–0.88)	0.84 (0.75–0.95)	0.86 (0.76–0.97)	0.78 (0.70–0.87)	0.92 (0.82–1.03)	0.94 (0.83–1.06)
<1000 (<846 USD)	0.69 (0.62–0.76)	0.81 (0.71–0.91)	0.83 (0.73–0.94)	0.63 (0.57–0.70)	0.83 (0.74–0.94)	0.86 (0.76–0.96)
Occupation						
Manager or professional	1.00	1.00	1.00	1.00	1.00	1.00
Clerk	1.02 (0.83–1.25)	1.12 (0.91–1.39)	1.15 (0.93–1.42)	0.87 (0.71–1.06)	0.90 (0.74–1.10)	0.90 (0.73–1.11)
Service or sales worker	0.89 (0.76–1.05)	1.00 (0.84–1.18)	1.02 (0.86–1.21)	0.77 (0.66–0.90)	0.91 (0.77–1.07)	0.92 (0.78–1.09)
Agricultural, forestry, or fishery worker	0.67 (0.58–0.78)	0.92 (0.78–1.08)	0.93 (0.79–1.10)	0.52 (0.45–0.60)	0.80 (0.68–0.94)	0.81 (0.69–0.95)
Mechanical or manual laborer	0.79 (0.68–0.91)	0.95 (0.81–1.11)	0.99 (0.84–1.16)	0.65 (0.57–0.75)	0.81 (0.69–0.94)	0.83 (0.71–0.97)
Housewife or student	0.95 (0.83–1.09)	1.20 (1.03–1.39)	1.15 (0.99–1.35)	0.70 (0.62–0.80)	1.00 (0.86–1.16)	0.96 (0.83–1.11)
National Basic Livelihood Security status						
Non-recipient	1.00	1.00	1.00	1.00	1.00	1.00
Recipient	0.97 (0.87–1.07)	1.09 (0.97–1.22)	1.08 (0.97–1.21)	0.89 (0.81–0.99)	1.03 (0.93–1.16)	1.02 (0.92–1.15)
Residence type						
Urban	1.00	1.00	1.00	1.00	1.00	1.00
Rural	0.72 (0.68–0.76)	0.83 (0.78–0.89)	0.86 (0.80–0.91)	0.66 (0.62–0.69)	0.77 (0.72–0.81)	0.79 (0.74–0.84)
Marital status						
Married	1.00	1.00	1.00	1.00	1.00	1.00
Divorced or separated	0.91 (0.80–1.03)	0.90 (0.79–1.02)	0.92 (0.80–1.05)	0.99 (0.88–1.12)	0.98 (0.87–1.12)	0.99 (0.87–1.13)
Widowed	0.80 (0.75–0.86)	0.87 (0.80–0.95)	0.91 (0.83–0.99)	0.71 (0.67–0.76)	0.84 (0.77–0.91)	0.86 (0.79–0.94)
Never married	0.86 (0.72–1.03)	1.02 (0.83–1.25)	0.99 (0.81–1.22)	1.02 (0.86–1.22)	1.05 (0.86–1.27)	1.01 (0.83–1.23)

Model 1: unadjusted. Model 2: adjusted for gender, age, and other socioeconomic factors. Model 3: adjusted for the same factors as Model 2 as well as smoking status, alcohol consumption, walking activity, perceived stress, diagnosis of hypertension, diagnosis of dyslipidemia, and receiving diabetes education.

Table 4 shows the associations between SES indicators and screening for DR and DN among subjects who had reported receiving diabetes education (n = 6,184). Overall, the results were similar to those described above. Those with a lower educational level and the lowest monthly household income, residents of rural areas, and widows/widowers had lower ORs for DR and DN screening. On the other hand, we found no significant association between occupation and DR or DN screening status.

**Table 4:** Relationships between screening for diabetic retinopathy and nephropathy and socioeconomic status indicators in subjects who reported receiving diabetes education (n = 6,184).

	Diabetic retinopathy screening	Diabetic nephropathy screening
Educational level		
College or higher	1.00	1.00
High school	1.02 (0.86–1.21)	0.95 (0.80–1.12)
Middle school	0.86 (0.70–1.05)	0.79 (0.65–0.97)
Primary school	0.78 (0.64–0.95)	0.81 (0.66–0.98)
No formal education	0.56 (0.44–0.71)	0.67 (0.53–0.85)
Monthly household income, thousand KRW		
≥5,000 (≥4,228 USD)	1.00	1.00
4,000–4,999 (3,383–4,227 USD)	1.04 (0.82–1.32)	1.03 (0.81–1.31)
3,000–3,999 (2,537–3,382 USD)	0.88 (0.71–1.09)	0.99 (0.80–1.23)
2,000–2,999 (1,691–2,536 USD)	0.90 (0.73–1.11)	1.00 (0.82–1.23)
1,000–1,999 (846–1,690 USD)	0.87 (0.71–1.07)	0.95 (0.77–1.16)
<1,000 (<846 USD)	0.76 (0.62–0.94)	0.81 (0.66–0.99)
Occupation		
Manager or professional	1.00	1.00
Clerk	1.08 (0.77–1.52)	0.90 (0.64–1.26)
Service or sales worker	1.05 (0.79–1.38)	0.96 (0.72–1.27)
Agricultural, forestry, or fishery worker	1.01 (0.76–1.34)	0.80 (0.60–1.05)
Mechanical or manual laborer	0.96 (0.74–1.24)	0.89 (0.68–1.15)
Housewife or student	1.25 (0.97–1.60)	1.03 (0.80–1.31)
National Basic Livelihood Security status		
Non-recipient	1.00	1.00
Recipient	1.10 (0.89–1.37)	1.13 (0.91–1.40)
Residence type		
Urban	1.00	1.00
Rural	0.85 (0.76–0.95)	0.86 (0.77–0.97)
Marital status		
Married	1.00	1.00
Divorced or separated	1.19 (0.94–1.50)	1.09 (0.87–1.38)
Widowed	0.83 (0.71–0.97)	0.83 (0.71–0.97)
Never married	1.06 (0.76–1.46)	1.08 (0.78–1.49)

Adjusted for gender, age, other socioeconomic factors, smoking status, alcohol consumption, walking activity, perceived stress, diagnosis of hypertension, and diagnosis of dyslipidemia.

## Discussion

We explored whether socioeconomic inequalities played roles in the frequency of DR and DN screening in a large population with diabetes. Educational level, monthly household income, occupation, residence type, and widow/widower status significantly affected the DR and DN screening frequencies. In particular, even among those who had reported receiving diabetes education, the DR and DN screening rates were significantly lower in those with less education and the lowest monthly household income, those in certain occupations, those residing in rural areas, and widows/widowers.

Epidemiological studies have revealed socioeconomic inequalities in diabetes care. A low SES is associated with poor metabolic control and a greater prevalence of diabetic complications, including DR and DN [17–20]. Moreover, low SES status is a powerful predictor of all-cause and cardiovascular mortality in type 2 diabetes patients, and socioeconomic disparities are not eliminated by controlling for conventional risk factors [21]. Socioeconomic status affects the extent of knowledge about diabetes, communication with healthcare providers, treatment choices, adherence to treatment, and access to medical care, social support, and community resources [18,22].

Education contributes to human capital by providing opportunities to acquire health knowledge, health literacy, problem-solving ability, and personal control [23]. More educated subjects tend to acquire better information and make informed choices with respect to lifestyle and health behaviors. Also, those with lower educational levels tend to lack the socioeconomic resources required for a healthy lifestyle, enjoy less social support, and not engage in networking. Consistent with previous studies [11,12,24,25], we confirmed that the frequency of DR and DN screening decreases as a function of educational level among diabetics, which is attributable to lack of knowledge, failure to understand the need to prevent diabetic complications, and lack of social resources and support. Thus, continuing public health efforts are needed to increase the screening rates for diabetic complications, especially in community-living diabetics with lower levels of education.

Previous studies on the associations between household income and DR and DN screening rates yielded inconsistent data. Some studies found that the lower the household income, the lower the DN screening rate, but no significant relationship was evident between household income and the DR screening rate [11,25]. However, in other studies, household income was not significantly associated with either the DR or DN screening rate [12,24]. We found that monthly household income was inversely associated with both the DR and the DN screening rate, although the OR for household income was not greater than that for educational level. Also, we found no difference in either the DR or the DN screening rate by NBLS recipient status (yes or no); this is an indicator of household economic status in Korea. We found that agriculture/forestry/fishery workers and machine/manual workers were screened for DN screening less frequently than were managers/professionals, but occupation and DR screening frequency were not significantly related. Only one prior study found a relationship between occupation and DR or DN screening frequency: service/sales workers, routine/manual workers, and unemployed individuals/housewives were screened less frequently for DR than were managers/professionals, but occupation did not significantly affect the frequency of DN screening [11].

As also found in previous studies [11,12], the DR and DN screening rates were lower in rural than in urban residents. Rural areas lack both ophthalmologists and primary physicians, and most medical facilities are located in urban areas. Indeed, the DR and DN screening rates differed significantly by residence type even among those who had reported receiving diabetes education. Thus, diabetes education alone is not enough to resolve the gap between urban and rural areas in the DR and DN screening rates. It is necessary to build more medical institutions, to expand community diabetes centers, to combine primary medical care with ophthalmology, and to introduce tele-ophthalmology [26–28].

Although marital status may not be an obvious socioeconomic indicator, it nonetheless influenced the frequency of screening for diabetic complications. Married subjects are supported by their spouses and tend to be more socially integrated, whereas bereavement removes the social, economic, and emotional support afforded by a spouse and weakens the social network [29,30]. Bereaved subjects engage in fewer health-promoting behaviors and use preventative health services less often than do married subjects [31,32]. We found that widows/widowers had significantly lower DR and DN screening rates compared with those who were married. However, few previous studies have specifically evaluated the relationship between marital status and screening for diabetic complications. More research is needed to determine the effect of marital status, especially bereavement, on screening.

As a lack of knowledge of the need to screen for diabetic complications is a major obstacle to seeking medical attention, diabetes education is crucial [33,34]. The absence of such education (rather than financial problems) significantly reduces the DR and DN screening rates [33]. In the present study, those who reported receiving diabetes education were 1.6- and 1.5-fold more likely to be screened for DR and DN than were those who had reported never receiving such education. Although 27.9% of subjects had reported receiving such education, socioeconomic inequalities (in terms of screening) remained even in such subjects. To prevent complications in community-dwelling diabetics, we need to adopt a multi-dimensional approach that includes active interventions targeting socioeconomic disparities as well as diabetes education.

Our study had several limitations. First, cross-sectional studies evaluate relationships, not causes and effects. Second, recall bias may have affected our results, as DR and DN test frequencies were self-reported. Furthermore, the accuracy with which respondents recalled DR screening (fundus photography), which is performed by an ophthalmologist, and DN screening (microalbuminuria), which is scheduled by a primary physician may differ. Third, all variables used in this study, including SES, health behaviors, and health status, were collected by questionnaire and were not confirmed or supplemented by examinations or measurements. Despite these limitations, the study has certain strengths. First, we used data from a national survey to access a large nationally representative sample of community-dwelling diabetics. We included several-fold more diabetics than any previous Korean study [11,12,24,25]. Second, we evaluated various socioeconomic factors that might affect the management of diabetic complications. Third, analysis of only those who had reported receiving diabetes education confirmed that socioeconomic inequalities were still in play even after such education. Indeed, certain fundamental socioeconomic inequalities are not eliminated by education.

## Conclusions

We identified socioeconomic inequalities affecting screening for DR and DN in a large population of diabetics. Diabetes education improved both DR and DN screening rates, but fundamental SES disparities remained. To reduce socioeconomic inequalities in screening, tailored interventions and societal support are needed for socioeconomically disadvantaged diabetics with low educational levels and low household incomes, those in certain occupations, those living in rural areas, and the bereaved.
